# Isolation of *Listeria monocytogenes* from poultry red mite (*Dermanyssus gallinae*) infesting a backyard chicken farm in Greece

**DOI:** 10.1038/s41598-023-27862-3

**Published:** 2023-01-13

**Authors:** Georgios Sioutas, Evanthia Petridou, Styliani Minoudi, Konstantinos V. Papageorgiou, Isaia Symeonidou, Ioannis A. Giantsis, Alexandros Triantafyllidis, Elias Papadopoulos

**Affiliations:** 1 Laboratory of Parasitology and Parasitic Diseases, School of Veterinary Medicine, Faculty of Health Sciences, Aristotle University of Thessaloniki, University Campus, 54124 Thessaloniki, Greece; 2 Laboratory of Microbiology and Infectious Diseases, School of Veterinary Medicine, Faculty of Health Sciences, Aristotle University of Thessaloniki, University Campus, 54124 Thessaloniki, Greece; 3 Department of Genetics, Development and Molecular Biology, School of Biology, Aristotle University of Thessaloniki, University Campus, 54124 Thessaloniki, Greece; 4 Department of Animal Science, Faculty of Agricultural Sciences, University of Western Macedonia, 53100 Florina, Greece

**Keywords:** Microbiology, Diseases, Risk factors

## Abstract

The poultry red mite (PRM), *Dermanyssus gallinae*, is arguably the most harmful, ubiquitous haematophagous ectoparasite infesting egg-laying hens. PRM is a vector of various microorganisms, with some being important for food microbiology and public health. The present study aimed to investigate the presence of specific pathogens, including *Escherichia coli*, *Salmonella* spp. and *Listeria* spp., carried by PRM infesting a chicken farm in Greece. Mites were caught using cardboard traps (Avivet), and 100 unwashed PRM were homogenized and used for microbiological cultures. Microbiological cultures were carried out on general and selective substrates to detect the above-mentioned bacteria. Specifically for *Listeria* spp., DNA was extracted from bacteria grown in Tryptone Soya Yeast Extract Agar using a commercial kit. The *hly* gene encoding the Listeriolysin O protein was amplified by PCR. Mites were identified as *D. gallinae* using morphological keys as well as by COI DNA barcoding. Microbiological cultures and PCR assays were positive for *Listeria monocytogenes*. No other bacteria were detected. The current study constitutes the first molecular isolation of *L. monocytogenes* from *D*. *gallinae*, confirming that PRM can carry this food-borne pathogen. PRM control measures and hygiene practices should be applied to minimize any possible contamination risk of poultry products with *L. monocytogenes* and safeguard public health.

## Introduction

The poultry red mite (PRM), *Dermanyssus gallinae* (De Geer 1778), is one of the most harmful ectoparasites in the modern egg-laying industry, having a worldwide distribution^1^. *D. gallinae* is an obligatory hematophagous mite and its blood-sucking feeding behaviour may negatively affect the welfare, health, and production of chickens^2,3^, effectively causing 231 million euros in losses solely in Europe^4^. Prevalences for the PRM are high in European laying hen farms^5^, even reaching 100% in Northern Greece^6^. Treatment options include synthetic pesticides, i.e., phoxim or fluralaner, and other biological or physical control measures^2^. However, PRM has developed resistance to different acaricides^7–9^ over the past decade, making its control even more difficult. Its life cycle consists of five stages. Eggs and the six-legged larvae do not feed on blood, while the eight-legged protonymphs, deutonymphs, and adults are hematophagous. Nymphs feed to moult to the next stage. Female adults feed to lay eggs, while male adults only feed periodically. Surprisingly, female mites can survive for 9 months without feeding^7^. Usually, the PRM feeds during the night, in darkness, for approximately 1 h every 2–4 days^1,8,9^. It prefers to feed on the hen’s body parts that are not covered with feathers, such as the breast and lower legs^10^, or from superficial veins on the neck and back^11^. Throughout the day, *D. gallinae* hides in the hens' environment, specifically cracks and crevices, under the egg belt or metal connections of cages, inside perches, or in the chickens’ nests^1^. They gain access to the host by travelling through the poultry house equipment and climbing up their legs or falling from the ceiling^11^. Unfed mites have a pale grey colour and, in contrast, engorged mites have a brown to bright red colour^12^. Adult females can drink 204 μg of blood, which amounts to 2.7 times their body weight^13^. In severe infestation cases, mites can ingest as much as 6% of the total blood volume of a hen within a day^9^. Under optimal conditions (30 °C temperature and 70–85% relative humidity) the life cycle can be completed in as little as six days^14^. As a result, population densities can increase rapidly, doubling in less than 6 days^15^, even reaching 150,000–200,000 mites per hen^16^. The PRM is more prevalent in summer than in winter, and mite populations reach their peak numbers around 5 months after the start of infestation before plateauing^17^. PRM of all stages are vulnerable to low (< 30%) relative humidity^7,18^ and are killed by washing the poultry houses^19^. Mites cannot withstand temperatures above 45 °C^20^ and below − 20 °C^7^. Extensive farming systems provide more hiding spots for the mites and make acaricide application difficult^21^. In addition, alternative systems and backyard farms exhibit higher PRM prevalence rates^22^.

Several pathogenic microorganisms have been isolated from the PRM including bacteria such as *Escherichia coli* and *Pasteurella multocida*^4^. For some of them, transmission and vector competence has also been demonstrated,, as is the case for Influenza type A virus^23^ and *Salmonella enterica* subsp*. enterica* serovar *Enteritidis*^24,25^. Some zoonotic pathogens are a major concern for food microbiology^4^, connecting many different disciplines of biological sciences such as parasitology, bacteriology and public health safety. For instance, the zoonotic bacterium *Listeria monocytogenes* is an emerging food-borne pathogen^26^ with reported listeriosis human cases^27^ and outbreaks^28^ usually attributed to contaminated poultry products instead of direct infections from infected chickens^29^. Contamination of poultry products (raw meat and eggs) might be caused directly by the hens or their environment^30,31^. Transmission can also occur through ingestion of contaminated water or airborne through contaminated soil and dust^29^. Clinical *Listeria* infections both in humans and in chickens are treated with antibiotics^31^. The connection between *L. monocytogenes* and *D. gallinae* up until now has been at the very least questioned^32^. *L. monocytogenes* has only been isolated once in a culture from PRM infesting wild animals and not chickens, more than 50 years ago with the original study presented in Russian and inaccessible to most researchers. Furthermore, no molecular tools were employed to confirm the pathogen’s identity^33^. Herein, we aim to report the first molecular detection of *Listeria* spp. in PRM. This finding is part of a larger study investigating the haplotypes of PRM and the presence of specific pathogens, including *Escherichia coli*, *Salmonella* spp. and *Listeria* spp., carried by PRM in 50 different backyard poultry farms in Greece. The current work could shed light on possible transmission routes of *L. monocytogenes* to chickens and consequently to humans through poultry products.

## Methods

### Backyard chicken farm history and sampling

The backyard poultry farm was located in Central Macedonia, Northern Greece and employed a free-range system. Sample collection was performed in October 2021. Prior to sampling, the farmer gave permission to take samples and filled in a questionnaire providing all appropriate information regarding farming practices. Based on the answers, the owner had more than 30 years of experience as a poultry farmer. Thirty-five hens of different ages were kept on the poultry farm, with most of them being 52 weeks old and belonging to the Lohmann brown breed. Even though PRM had been infesting the farm for many years, the owner believed that PRM did not affect hen health and that egg production was on the expected level. However, the chickens only produced eight eggs per day, despite their age. All hens were vaccinated with commercial vaccines against *Salmonella* spp. and Marek's disease virus. Mites were visible on the walls (flat surfaces), but the eggs did not have any blood spots (from crushed mites). Moreover, the owner complained of feeling PRM bites on the skin and itching.

The hen house was primarily made of wood with some bricks. On the outside, trees with other birds’ nests, such as swallows and pigeons, surrounded it. Chickens roamed freely in the farm's backyard and sometimes fed on the owner's home-grown vegetables. The owner did not use any egg cartons but instead collected the eggs in a bucket. Chickens did not receive any treatment for PRM, such as fluralaner, deltamethrin, diatomaceous earth, desiccant dust, or other formulations. Only cold water was used once every 3 months to wash the hen house, but with no visible effect on PRM populations. Furthermore, no monitoring devices such as cardboards were employed to assess fluctuations in PRM numbers. In order to catch the PRM, ten specially designed cardboard traps, the AviVet Red Mite Trap™, (Avivet, adVee Dierenartsen, Heeswijk Dinther, The Netherlands) were used^34^ that were placed inside various cracks, perches, and nests, where mites usually hide during the day^1^. The traps covered the entirety of the hen house and were left for one week before being collected again to maximise the number of PRM caught. Consequently, traps were placed inside plastic sealed bags and transported to the School of Veterinary Medicine, Faculty of Health Sciences, Aristotle University of Thessaloniki for further examination.

### Mite identification

A few drops of lactophenol were used to soften and clarify the mites before examining them at 100 × and 400 × magnification under an optical microscope (Olympus, CX21 Microscope). The genomic DNA of three separate mites was extracted using a commercial kit (QIAamp DNA mini kit Extraction Kit, Qiagen, Hilden, Germany) as previously described^35^. The synthetic oligonucleotide primers COI1Fyuw114 and COI1Ryuw114^35^ were used in this study to amplify a partial mitochondrial Cytochrome C Oxidase subunit I (COI) gene segment, 681 base pairs (bps) in length. PCR reaction was carried out in a 30 μL volume comprising 3 μL of 10 × Buffer (Qiagen), 1.2 μL (2.5 mM) MgCl2 (Qiagen), 0.3 μL (100 µM) of each oligonucleotide primer, 0.75 μL (2 mM) dNTPs, 0.45 μL (0.05 U) of Taq polymerase (Qiagen) and 5 μL (100 ng) of genomic DNA. PCR amplification was achieved using an Eppendorf Mastercycler (Eppendorf, Hamburg, Germany) with the following cycling program: initial denaturation (5 min, 95 °C), 35 denaturation cycles (30 s each, 95 °C), annealing (45 s, 58 °C), and extension (40 s, 72 °C) prior to a final extension cycle (7 min, 72 °C). Both positive and negative controls were employed, and the resulting PCR products were analysed by agarose gel electrophoresis (AppliChem, Darmstadt, Germany) and visualised using ultraviolet light. Amplicons purification, sequencing and alignment of the derived sequences were carried out as described in a previous work of our lab^35^.

### Bacterial cultures

Microbiological cultures were performed for *E. coli*, *Salmonella* spp. and *L. monocytogenes* using selective media. In detail, *L. monocytogenes* was isolated from the samples using the methodology based on EN ISO 11290-1:2017. Approximately 100 unwashed bodies of *D. gallinae* mites were pooled from the traps and homogenised in 0.9 ml of half Fraser broth (Biolife, Milan, Italy) using Biomasher II disposable homogeniser tubes (Nippi Inc, Tokyo, Japan) and incubated at 30 °C for 24 h. For the secondary enrichment, 0.1 ml of the culture was transferred to a tube containing 10 ml of Fraser broth (Biolife) and incubated at 37 °C for 24 h. Consequently, a loopful (10 μl) from Fraser broth was streaked onto Agar Listeria acc. to Ottaviani and Agosti (ALOA) (Biolife) and *Listeria* Palcam agar (Biolife) (37 °C, 48 h). Five suspected *L. monocytogenes* colonies were streaked on Tryptone Soya Yeast Extract (TSYE) agar (Merck, Darmstadt, Germany) (24 h, 37 °C) for conducting the confirmation tests (Beta-haemolysis, L-Rhamnose, D-Xylose).

### DNA extraction from cultures

DNA was extracted from bacterial colonies grown in TSYE agar using the Nucleospin Tissue extraction kit (Macherey–Nagel, Duren, Germany). In detail, bacterial colonies were removed from TSYE Agar plates and suspended in 1 ml phosphate-buffered saline (pH 7.4). Consequently, 0.2 ml of the above suspension was used for DNA extraction following the instructions of the selected kit.

### PCR assay and primers for *L. monocytogenes*

The synthetic oligonucleotide primers (working solution 10 μM) used in this study and the size of the amplified selected fragment are listed in Table 1^36^. This primer pair amplifies a species-specific PCR product in *L. monocytogenes*, whereas no product is amplified in other *Listeria* spp. or other bacterial genera) and therefore can reliably distinguish the presence of *L. monocytogenes*^37^.

**Table 1:** Nucleotide sequences of primer sets used in this study.

Gene target	Primer sequence (5’–3’)	Product size (bps)	Protein encoded by the target gene
*Hly*	LL5: AAC CTA TCC AGG TGC TC	520	Listeriolysin O (LLO)
	LL4: CGC CAC ACT TGA GAT AT		

The amplification was performed in a total volume of 20 μL containing 2 μL of DNA sample, 1.5 mM MgCl2, 0.2 mM (each) dNTPs, 0.2 μM of each primer, and 0.2 U/reaction of Taq DNA polymerase (KAPA Biosystems, Germany). PCR assays were performed with a model T100 thermal cycler (Bio-Rad, California, USA) under the following conditions: 95 °C for 3 min and then 35 cycles consisting of denaturation at 95 °C for 30 s, annealing at 55 °C for 30 s, and extension at 72 °C for 1 min with a final extension step at 72 °C for 7 min. Five microliters of the reaction mixture were mixed with 2 μl of loading buffer and separated on a 1.5% agarose gel in a TBE buffer (90 mM Trizma base, 90 mM boric acid, 2 mM EDTA, pH 8.3). The PCR product was visualised by ethidium bromide staining on a UV transilluminator (Cleaver Scientific Ltd, Warwickshire, UK).

This research was carried out under the approval from the Ethics Committee of the Aristotle University of Thessaloniki (639/13-07-2020). All experiments were performed in accordance with relevant guidelines and regulations. Informed consent was obtained from all the participants to participate in this study. Also, the farmer gave permission to publish any relevant information arising from the study.

### Ethics approval and consent to participate

There was no interaction with the chickens or harm caused to them. This research was carried out under the approval from the Ethics Committee of the Aristotle University of Thessaloniki (639/13-07-2020).

## Results

### Mite identification results

In the Laboratory of Parasitology and Parasitic Diseases, mites were identified as *D. gallinae* based on morphological criteria^38^. The resulting PCR products from the 3 separate mites were 681 bps long based on gel electrophoresis and transillumination. DNA sequencing was successful in all 3 individual mite samples, and all 3 mites had one identical haplotype that was 520-bps long (GenBank accession number: ON597616). The haplotype identified in the current study was 99.81% similar to other PRM haplotypes previously identified in Japan^39^, further confirming that mites belonged to the species *D. gallinae*.

### Bacterial cultures and PCR for *L. monocytogenes*

**Figure 1:**
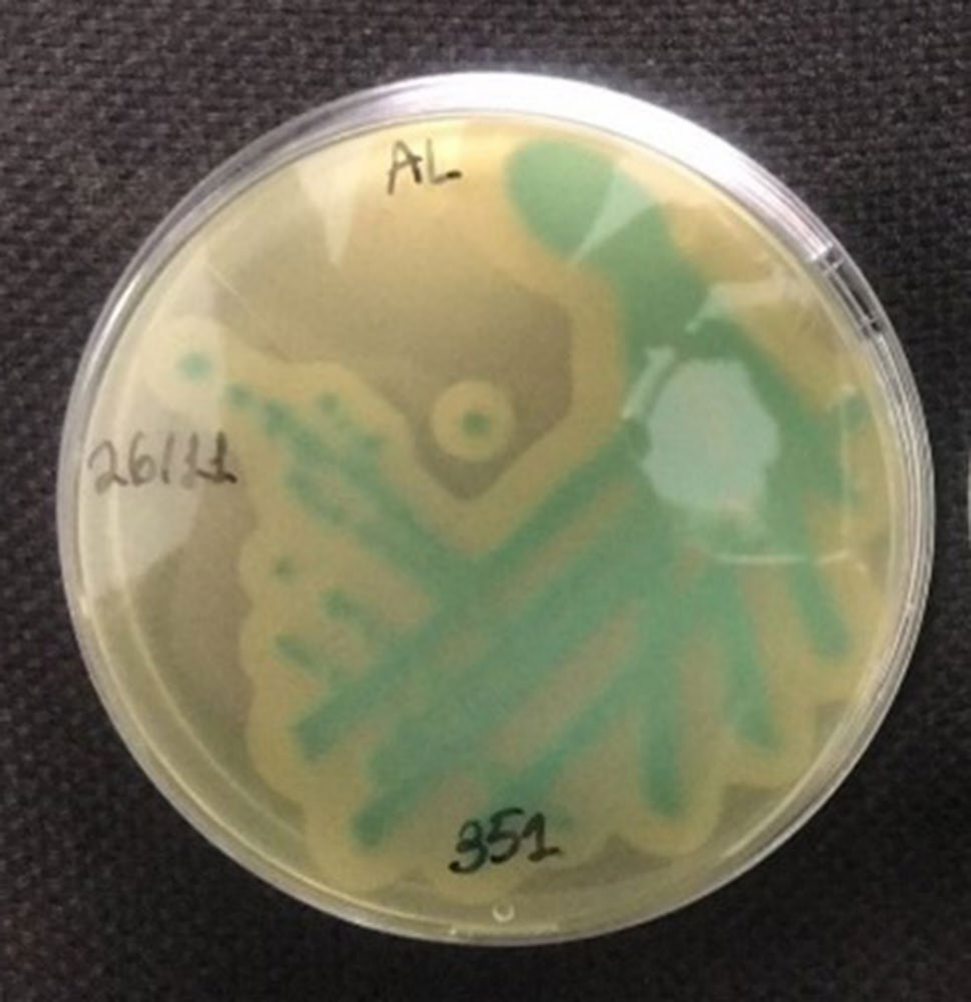
*Listeria monocytogenes* colonies after cultivation on Tryptone soya yeast extract (TSYE) agar.

**Figure 2:**
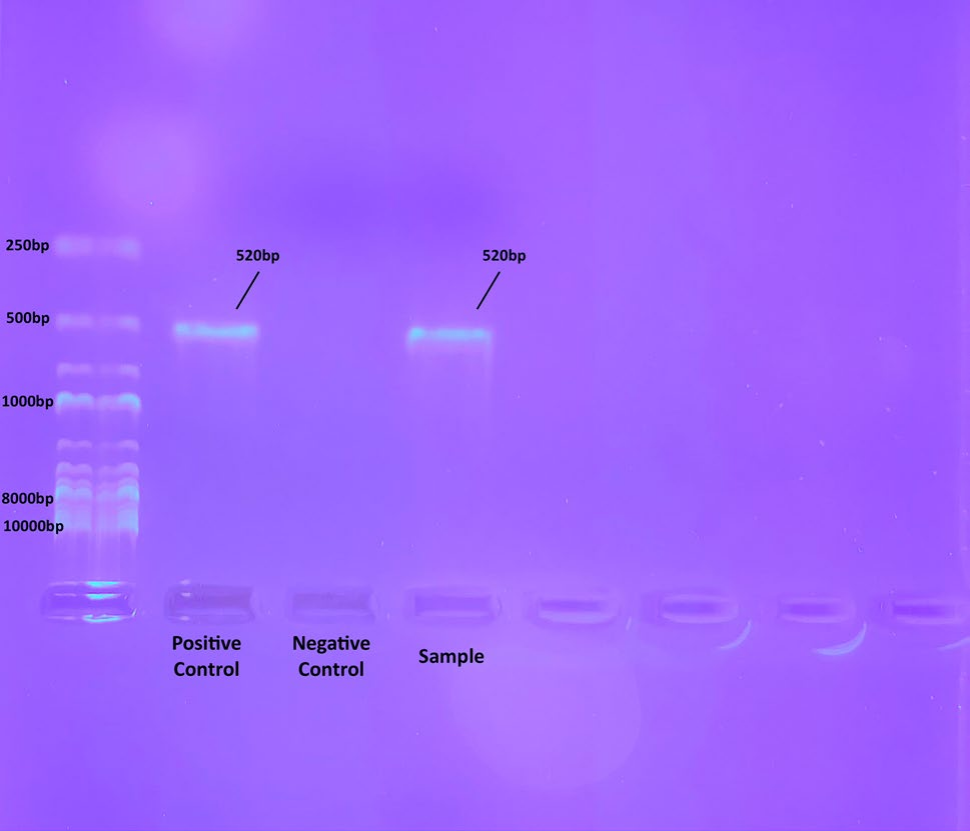
Transillumination under ultraviolet light of the 520 base pairs (bps) long PCR product after agarose gel electrophoresis and ethidium bromide staining, confirming the presence of *Listeria monocytogenes* on *Dermanyssus gallinae*. The sampled DNA was extracted from bacterial colonies grown in TSYE agar, and the primers used targeted the hly gene that encodes the Listeriolysin O protein. *Abbreviations:* PCR, Polymerase chain reaction; TSYE, Tryptone soya yeast extract.

Microbiological cultures and confirmation tests were only positive for *L. monocytogenes* (Fig. 1) and negative for *E. coli* and *Salmonella* spp. DNA was successfully extracted from TSYE agar and amplified using PCR. The resulting PCR product was 520 bps long based on gel electrophoresis and transillumination (Fig. 2), further confirming the presence of *L. monocytogenes*.

## Discussion

*L. monocytogenes* is a ubiquitous Gram-positive facultative pathogenic saprophyte with an intracellular life cycle^40^ that can persist in the environment at temperatures between 0 °C and 45 °C^31^. It can replicate under refrigerator conditions^31^ and survive in high moisture environments for many years^29^. *L. monocytogenes* is the primary aetiologic agent of listeriosis, an infectious disease affecting humans and birds, among other animals^26,31^. Although most bird infections are subclinical and adult chickens rarely display clinical signs, listeriosis outbreaks have been reported in backyard poultry farms^41^. Young chicks are typically more susceptible^29^, and clinical signs include diarrhoea, encephalitis, septicaemia, lower egg production^41^, and mortality^29^. Like the sampled farm in the current study, chickens living in humid and low-temperature environments with wet litter have a higher risk of infection^29^. Other predisposing risk factors that can increase environmental contamination with *L. monocytogenes* at the farm level include lack of sanitary measures, no vermin control programs, use of nipples without cups as a watering system, presence of other animals at the farm, and inadequate disposal of faeces^42^. Noteworthy, the current backyard farm sampled had all these risk factors.

The bacterium consists of four distinct lineages^40^ and, until now, 14 serotypes have been identified^43^. Human listeriosis cases can be primarily attributed to lineage I and secondarily to lineage II, with the latter mainly being isolated in food and food production facilities^40^. Listeriolysin O (LLO) is a cholesterol-dependent cytotoxin found in *L. monocytogenes* and is encoded by the *hly* gene^44^. The LLO peptide significantly increases the strain's virulence^45^ and is linked with human listeriosis outbreaks^44,46^, although other genes have also been reported to affect virulence^31^. LLO-positive strains are 5-logs more virulent and spread more quickly than LLO-negative strains^46^. PCR is a fast and sensitive technique that can amplify the *hly* gene to confirm the presence of *L. monocytogenes* from selective growth media^29^, as carried out in the present study. Determination of the infecting lineage and serotype is vital in human infections in the One Health approach^47^ since listeriosis is an emerging food-borne zoonotic disease^29^. Virtually all *L. monocytogenes* infections are food-borne^40^. Chickens with listeriosis can infect humans, and the disease can result in the patient's death in just two days^29^. Humans can also exhibit cutaneous lesions after direct contact with infected chickens or contaminated soil^29^. Contamination of poultry products, mainly chicken carcasses, is caused by poor hygiene measures and unsafe handling practices, i.e., not washing hands or cutting boards and not separating raw and cooked meat^27,48^. In our study, the specific *L. monocytogenes* strain was positive for the LLO protein, based on the successfully amplified *hly* gene.

*D. gallinae* in the specific poultry farm probably acquired *L. monocytogenes* when moving inside the poultry house^49^. Chickens can serve as natural reservoirs for the specific pathogen^50^ and excrete *L. monocytogenes* with their faeces^51^ and other secretions^29^. The farm’s environment, such as dust^52^, litter^53^, soil^31^, grass^54^, water, feed^55^, decomposing vegetation^41^, nests, walls, floors, faeces, and other matrices can be contaminated with *L. monocytogenes*^29,30^. Transmission occurs when chickens ingest these contaminated sources, when their wounds get contaminated or when they inhale the pathogen^29^. *L. monocytogenes* is common in poultry in Greece and, in the past, has been isolated from 38% of poultry samples in a slaughterhouse^56^. Since mites were not washed before performing the bacteriological culture, we cannot distinguish if *L. monocytogenes* was harboured inside the PRM or just mechanically carried outside on its cuticle. In our study, the mites were processed according to published methodology (without washing them)^57–59^, though in some other studies mites were washed for example with 4% paraformaldehyde^60,61^. Mites were internally infected or externally contaminated (i.e., in their dorsal shield, genitoventral shield, legs, and chelicerae)^38^ from apparently healthy chickens or other environmental sources. As demonstrated in previous studies, not washing the PRMs before processing provides more information and all-round knowledge on the vectorial potential of *D. gallinae*^59^. According to our results, the PRM can be added to the list of *L. monocytogenes* vectors, alongside carriers such as rodents, insects, and flies that can disseminate the pathogen on a farm^29^. *D. gallinae* can actively or passively move between facilities (i.e., poultry houses and slaughterhouses) using humans as transport hosts (hair, shoes, clothes) or through tools and equipment (egg containers, pallets, crates, and brooms, among others)^19^. In this backyard farm, chickens were mainly kept for eggs, but occasionally, some were slaughtered for their meat. The farm also had no sanitary measures in effect. Therefore, *Listeria*-carrying *D. gallinae* could suck blood from chickens and infect their wounds or get eaten by them, which represent the two most common pathogen transmission routes from PRM to hens^62,63^. Moreover, the farm had a heavy PRM infestation, as evident by the mite populations caught in the traps and mites walking on equipment that could significantly enhance the likelihood of mechanical transmission^64^ and help spread *L. monocytogenes*. Contamination of poultry products was a real possibility that could threaten public health^65^. Suggestions were made to the farmer regarding disinfection of the farm for *L. monocytogenes* and treatment of hens with a licensed product for PRM.

Future research should focus on elucidating possible transmission routes of *Listeria* spp. Between mites and hens and detecting the same genotype in mites and meat after slaughter. That should be done on much more numerous samples (different timepoints and farms) to assess the PRM vectorial role for the specific pathogen. Nevertheless, the current work constitutes a preliminary study that helps to solidify the broad spectrum of *D. gallinae* as a vector of different pathogens.

## Conclusions

The current work describes the first molecular isolation of *L. monocytogenes* from PRM, confirming that *D. gallinae* can carry this food-borne pathogen, which has only been questionably isolated once in the past. Control measures are required to reduce PRM populations in chicken farms, and farmers should additionally apply hygiene and sanitisation practices to minimise any possible contamination risk of poultry products with *L. monocytogenes*. Since antibiotics are used to treat listeriosis in hens and humans, combating *D. gallinae* in poultry houses could prevent transmission of *L. monocytogenes*, and reduce the need for antibiotics while protecting public health. The finding of *L. monocytogenes* further expands the vectorial role of *D. gallinae* highlighting how the PRM could impact other sectors, outside the sampled farm, such as food production facilities threatening public health safety. Overall, the PRM may severely affect hen health both through its haematophagous action and its ability to transmit pathogens.

## Data Availability

The nucleotide sequence data of the 520-bps long identical haplotype identified in all 3 individual mite samples has been deposited in GenBank (GenBank Accession Number: ON597616).

## Abbreviations

Bps: Base pairs
COI: Cytochrome oxidase subunit 1
DNA: Deoxyribonucleic acid
LLO: Listeriolysin O
PCR: Polymerase chain reaction
PRM: Poultry red mite
TSYE: Tryptone soya yeast extract
